# Abnormal Admission Chest X-Ray and MEWS as ICU Outcome Predictors in a Sub-Saharan Tertiary Hospital: A Prospective Observational Study

**DOI:** 10.1155/2016/7134854

**Published:** 2016-09-19

**Authors:** Hannington Ssemmanda, Tonny Stone Luggya, Clare Lubulwa, Zeridah Muyinda, Pascal Kwitonda, Humphrey Wanzira, Joseph Ejoku

**Affiliations:** ^1^Directorate of Surgery Surgical Services, Mulago National Referral Hospital, Kampala, Uganda; ^2^Department of Anaesthesia, Makerere University College of Health Sciences, Kampala, Uganda; ^3^Department of Radiology, Mulago National Referral Hospital, Kampala, Uganda; ^4^Department of Epidemiology, Ministry of Health Uganda, Kampala, Uganda

## Abstract

*Background*. Critical care in Uganda is a neglected speciality and deemed costly with limited funding/prioritization. We studied admission X-ray and MEWS as mortality predictors of ICU patients requiring mechanical ventilation.* Materials and Methods*. We did a cross-sectional study in Mulago Hospital ICU and 87 patients for mechanical ventilation were recruited with mortality as the outcome of interest. Chest X-ray results were the main independent variable and MEWS was also gotten for all patients.* Results*. We recruited 87 patients; most were males (60.92%), aged between 16 and 45 years (59.77%), and most admissions for mechanical ventilation were from the Trauma Unit (30.77%). Forty-one (47.13%) of the 87 patients died and of these 34 (53.13%) had an abnormal CXR with an insignificant IRR = 1.75 (0.90–3.38) (*p* = 0.062). Patients with MEWS ≥ 5 (*p* values = 0.018) and/or having an abnormal superior mediastinum (*p* values = 0.013) showed a positive association with mortality while having a MEWS ≥ 5 had an incidence risk ratio = 3.29 (1.00–12.02) (*p* = 0.018). MEWS was a good predictor of mortality (predictive value = 0.6739).* Conclusion*. Trauma (31%) caused most ICU admissions, having an abnormal admission chest X-rays positively associated with mortality and a high MEWS was also a good predictor of mortality.

## 1. Background

In Uganda, critical care is a neglected speciality especially in government hospitals where there is evidence of a dire lack of Intensive Care Unit (ICU) beds while the private hospitals, located in the capital Kampala, with the most ICU beds are exorbitantly expensive for the average Ugandan to afford. Most patients therefore seek ICU care at Mulago National Referral and Teaching Hospital (MNRTH), a tertiary healthcare institution already stretched beyond its initially designed 1500-bed capacity and a mortality rate of 40.1% mainly due to septic patients with head injury, acute lung injury, and HIV/AIDS [1]. The nonprioritization of critical care in resource limited settings is possibly due to a rechannelling of most resources towards infectious diseases like HIV/AIDS, malaria, and tuberculosis, which has led to unavailability of ICU establishments in most of the hospitals [2]. In contrast, developed countries like the USA have well established ICU settings in almost all hospitals where 55,000 patients are cared for daily in 6,000 ICUs [3].

There is already evidence that critical care improvement can be achieved in a resource limited setting with Kenya, to the east of Uganda, which has established ICU as a basic component of their district health units [4]. This example contradicts the notion that critical care is defined by expensive technology [5], making the lack of its prioritization unjustifiable since virtually all hospitals have critical patients especially since in Uganda we have a predominantly youthful population of 95% aged 0–54 and 48% aged 0–14 years [6]. With this background, it is necessary to find a cheap and routinely usable method to predict mortality in ICU establishment, with the aim of prioritizing treatment to those with a poor prognosis.

In Mulago, the most common indication for ICU admission was respiratory support with a majority requiring invasive mechanical ventilation; however, none of these patients received an admission CXR yet they had a higher mortality with an odds ratio of 4.53 [7]. Chest X-rays (CXRs) in Mulago are readily available with studies showing they aid in the early detection of pulmonary abnormalities translating in better outcomes plus a reduced length of ICU stay [8]. CXRs in the ICU also reveal significant pathology in 65% admitted patients and this has resulted in changed patient management [9]. Additionally, in the intensive care units, predictive scoring systems aid in measurement of disease severity and prognosis of patients and are helpful in clinical decision-making, standardizing research, and comparing the qualities of patient care across ICUs [10]. This study thus sought to determine if an abnormal chest X-ray finding was a predictor of mortality among patients that required mechanical ventilation in Mulago Hospital general ICU. The study also assessed whether the Modified Early Warning Score (MEWS) on admission had an association with mortality among a similar study population.

## 2. Materials and Methods

We obtained ethical approval from the Department of Anaesthesia and Critical Care, MNRTH, and Makerere University's College of Health Sciences Institutional Review Board at the School of Medicine Research and Ethics Committee (SOMREC) to conduct a prospective descriptive study from February to December 2014. Mulago National Referral Hospital Intensive Care Unit was the study site with a 1,500-bed capacity and a 12-bed ICU able to invasively ventilate 6 patients at a time. It receives technical support from the Department of Anaesthesia and is staffed with 7 doctors (5 anaesthesiologists and 2 physicians) with 20 nurses. The average nurse to patient ratio is 1 : 4 and about 240 annual admissions with half of this requiring mechanical ventilation. The ICU offers level II care which includes mechanical ventilation for longer than 24 h and specific organ support like dialysis and inotropic infusions. It can also provide mechanical ventilation, postoperative care, intermittent haemodialysis, peritoneal dialysis, and basic neurocritical care [1] but it is not equipped with neonatal and infant ventilators. Due to Mulago Hospital's planned reconstruction and relocation of ICU [11, 12], its admission capacity during this study had dropped to 6–8 patients with the ability to ventilate only 4 patients.

### 2.1. Study Procedure

An internal review board waiver of consent was granted for this study and we included all patients with an admission X-ray within 24 hours and excluded those with poor CXR quality or failure to get an X-ray within 24 hours.

Upon admission for mechanical ventilation, the patients got an admission CXR which was analysed by two independent radiologists both blinded to the others' interpretation. Additionally, the admission MEWS was assessed as a predictor of mortality among patients on mechanical ventilation. After patient disposition from the ICU, the length of stay and final outcome of the patients were noted.

### 2.2. Study Variables

The main outcome of the study was mortality and the main independent variable was chest X-ray status (normal or abnormal). Other independent factors considered included gender, MEWS, costophrenic recess status, hilar, heart shadow and superior mediastinum, and presence of pleural fluid. Additionally, we reported results of validity (sensitivity and specificity) of MEWS in predicting mortality.

### 2.3. Sample Size Calculation

The proportion of abnormal CXR was based on Henschke et al. [9]'s study where approximately 65% of routine CXR showed an abnormality. Using Kish and Leslie formula confidence interval (*Z*) of 95%, with a chosen precision of 10% and *Z* being 1.96, we derived a sample size of 91 after factoring in loss to follow-up (5%).

### 2.4. Data Management and Analysis

A structured questionnaire was used with radiologist reports retrieved and entered for each study participant. Data was cleaned, coded, entered into Epidata version 3.1, and then analysed. Stata version 12 (Statcorp, College Station, Texas, USA) was used for all analysis. The distributions of study participant baseline characteristics were presented as frequencies with respective proportions. A Poisson regression model was used to assess categorical factors associated with mortality between patients with normal and those with abnormal chest X-rays to derive the adjusted risk ratio with its respective confidence interval. A *t*-test was used to estimate the difference in mean ICU length of stay between those with abnormal and normal X-rays. Kaplan-Meier analysis was used to estimate the probability of survival between the two X-ray arms and a log-rank test was used to assess the level of significance of the differences in probability. In all analysis *p* value < 0.05 was considered as statistically significant. ROC analysis was performed to determine the area under the curve and establish whether MEWS was a predictor of mortality.

## 3. Results

### 3.1. Participants' Characteristics

**Figure 1:**
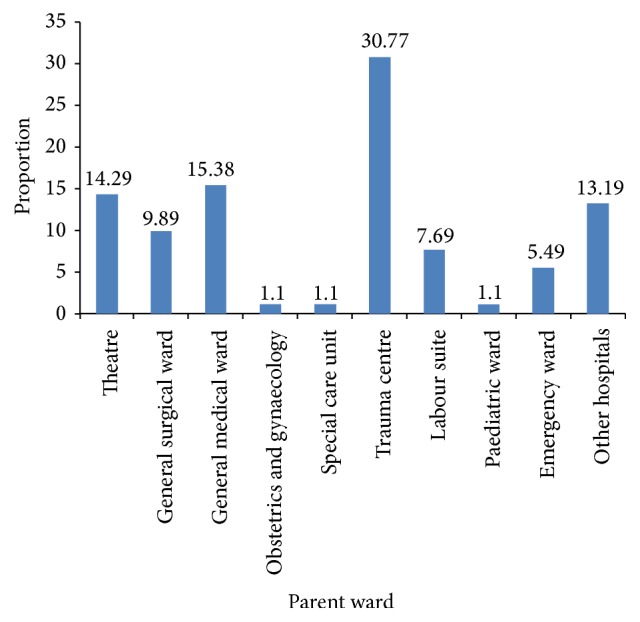
Graph of admissions to ICU from parent ward.

**Figure 2:**
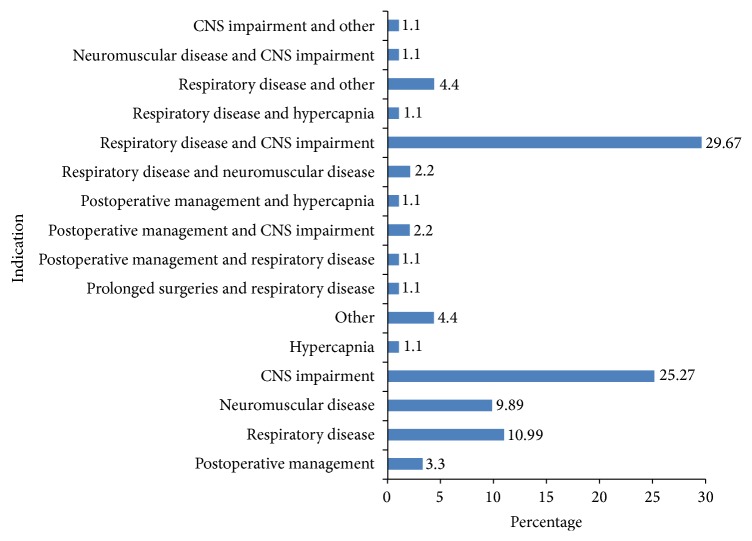
Indication of mechanical ventilation.

We screened 91 patients and enrolled 87 to the study, with 4 participants excluded due to poor quality of the CXR which was difficult for the expert radiologist to interpret. Most patients were aged 16–45 years (59.8%) with 53 males (60.9%) and 34 females (39.1%). At admission, 73 (84.9%) patients had MEWS ≥ 5 and 14 (15.1%) had a MEWS < 5 (see Table 1). Most admissions for mechanical ventilation came from the trauma centre (30.8%) and this was followed by general medical ward, theatre, and external referrals labour suit as shown in Figure 1. CNS impairment (25.3%) associated with respiratory dysfunction was the commonest indication for mechanical ventilation mainly due to low GCS (see Figure 2). Radiologists' assessments showed normal lung parenchyma for 28.7% and pathology in 71.3% with 23% of these showing a picture consistent with opacities indicative of infection followed by interstitial lung disease (16%) and alveolar lung disease (11%).

### 3.2. Mortality and Factors Associated

**Figure 3:**
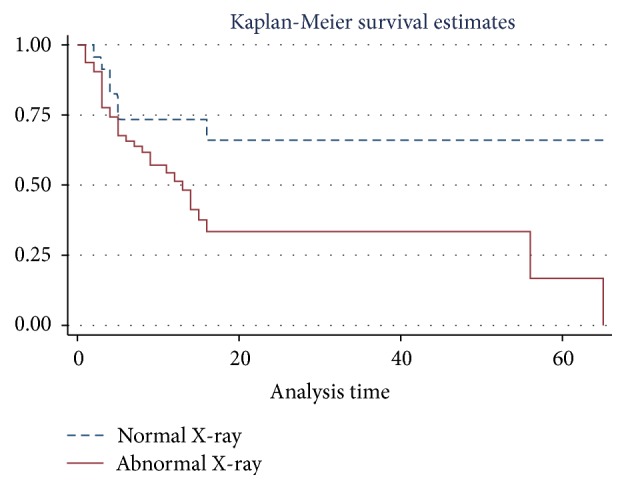
Survival analyses based on chest X-ray findings.

Forty-one (47.1%) of the 87 patients died and of these 34 (53.1%) had an abnormal CXR (*p* = 0.064) with an insignificant incidence risk ratio of 1.75 (*p* = 0.06). Patients with MEWS ≥ 5 (*p* values = 0.02) and/or having an abnormal superior mediastinum (*p* values = 0.01) showed a positive association with mortality. Even though an abnormal chest X-ray finding was associated with nearly double the mortality (IRR = 1.75 [0.90–3.38]), this was not statistically significant (*p* = 0.06) (see Table 2). Patients with abnormal chest X-rays on average had shorter stay in the ICU (11.1 days) compared to those with normal CXR (16.73 days); also the probability of survival was higher among those with a normal chest X-ray when compared to those with an abnormal X-ray and this was statistically significant (*p* = 0.03) as shown in Figure 3.

### 3.3. Predictive Value of CXR and MEWS of Mortality at ICU

**Figure 4:**
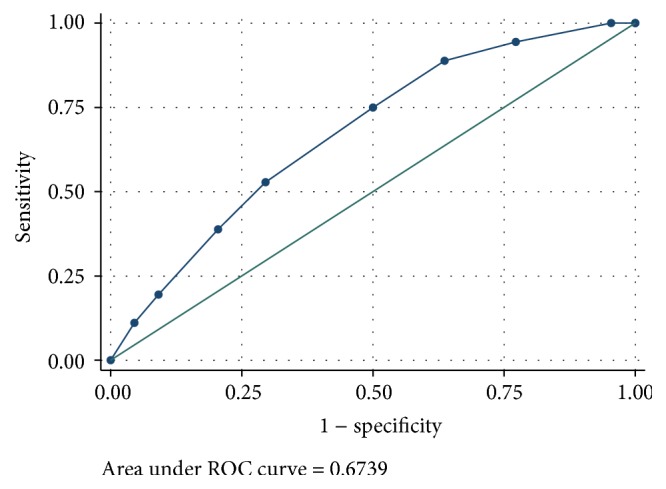
Area under the curve for MEWS.

An abnormal X-ray was 82.93% sensitive and 34.78% specific in predicting mortality (see Table 3), while MEWS with a cut-off of ≤5 had a sensitivity of 5.13% and specificity of 76.60% of predicting mortality. When the ROC curve was plotted, the MEWS was a poor predictor of mortality with 0.6739 area under the curve graph (see Figure 4).

## 4. Discussion

This study has found that males were the majority of admitted patients for mechanical ventilation at the ICU with most admissions between 16 and 45 years. Trauma was the commonest need for ICU admission which signifies its persistent burden to Mulago Hospital as other studies showed trauma associated with severe head injury was the most common comorbidity for cardiac arrests in Mulago Hospital [13]. This glaring burden will remain persistent due to lack of critical care prioritization and facilities yet prior studies have shown Uganda had the world's second highest accident burden of over 20,000 road accidents annually, with 2,334 fatalities [14].

Having an abnormal admission chest X-rays may positively predict mortality among patients admitted to the ICU for mechanical ventilation; however, this prediction is not conclusive given its low specificity. Indeed, there is evidence of an association of an abnormal chest X-ray with mortality, with the former nearly doubling mortality in this population, but this was not statistically significant, possibly due to the small sample size of the study. Additionally, the probability of mortality of patients with an abnormal chest ray was significantly higher than those who had a normal chest X-ray. Even though some relatively equipped hospital departments currently use innovations like Picture Archiving and Communicating System for their imaging, however Mulago continues to use CXR films [15]. Although the evidence so far does not clearly support the use of chest X-rays to predict mortality, there is an encouraging tendency towards this prediction with study limitations notwithstanding. X-rays are cheap and available and aid in the early detection of cardiopulmonary abnormalities which can be protocolized in low resource settings in line with standards of practice because [9], also, if utilized routinely they can identify radiographic abnormalities that can lead to a change in treatment [16, 17], translating into better mortality outcomes with reduced length of hospital stay. Despite their interpretation being subject to an individual's experience [18], they are paramount in the ICU because clinicians opt for them routinely or only conservatively when needed depending on unit protocols [19–22]. Also as much as many CXRs may not disclose new findings, they have a substantial impact on the management of intubated and mechanically ventilated patients in the ICU [22, 23], for example, their poor sensitivity in the diagnosis of pulmonary embolism, yet they aid in ruling out other pathologies that may have a clinical presentation similar to that of pulmonary embolism [24].

Additionally, a MEWS of ≥5 was significantly associated with higher mortality in Mulago Hospital ICU, which was moderately supported as a predictor of mortality on analysis of AUC graph with an area under the curve at 0.67, a result that is comparable to other ICU studies that showed it was a validated assessment tool for detecting risk of deterioration in spontaneously breathing patients in the ICU [25]. Therefore, a MEWS can be used to predict mortality, as it allows prompt communication between nursing and medical staff when patients' condition deteriorates, thus enabling early admission, making it easy to implement, and an important risk management strategy [26].

Our study limitations included system issues like hospital reconstruction and portable X-ray machine not being available all the time that affected patient recruitment; we did not assess laboratory parameters that may have had an effect on mortality due to diversity of pathology admitted yet studies have shown they play a part in prediction [27].

Our study findings suggest the use of admission CXRs and MEWS as a routine parameter in our resource limited setting to aid in predicting mortality in ventilated patients and this has been shown to hold true in other studies [8, 9, 28]. This can be adopted as critical care relies on the stepwise introduction of service improvements, leveraging human resources through training, a focus on sustainable technology, ongoing analysis of cost-effectiveness, and the sharing of context-specific best practices [29]. Even though other studies reported significant evidence to support the use of chest X-rays in predicting mortality, our study has shown this to be moderate and we recommend larger studies to be done to assess this further.

## 5. Conclusion

Our findings showed that trauma is the highest cause of ICU admissions in Mulago and having an abnormal admission chest X-rays may positively be associated with mortality while a higher MEWS is a good predictor of mortality in patients with mechanical ventilation admitted to the ICU. These can serve as predictors of early lifesaving interventions or mortality in low resourced ICUs; however, larger studies evaluating further CXR evidence are recommended.

## Figures and Tables

**Table 1:** Distribution of study participants' characteristics.

Characteristic	Number	Percentage
Age categories (years)		
≤5	7	8.05
6–15	10	11.49
16–45	52	59.77
>45	18	20.69

Gender		
Male	53	60.92
Female	34	39.08

MEWS		
<5	14	15.12
≥5	73	84.88

**Table 2:** Mortality risk factors in ventilated patients.

Variable	Outcome status		IRR (95% CI)	*p* value
	Dead	Alive		
Gender			1.39 (0.91–2.13)	0.135
Male	23 (53.49)	33 (68.75)		
Female	20 (46.51)	15 (31.25)		

MEWS			3.29 (1.00–12.02)	0.018
<5	2 (5.13)	11 (23.40)		
≥5	37 (94.87)	36 (76.60)		

Chest X-ray status			1.75 (0.90–3.38)	0.062
Normal	7 (17.07)	16 (34.78)		
Abnormal	34 (82.93)	30 (65.22)		

Costophrenic recess			1.27 (0.77–2.11)	0.387
Normal	31 (77.50)	39 (84.78)		
Obscured	9 (22.50)	7 (15.22)		

Pleural fluid			0.83 (0.38–1.84)	0.631
Absent	37 (90.24)	40 (86.96)		
Present	4 (9.76)	6 (13.04)		

Hilar			1.49 (0.88–2.50)	0.215
Normal	35 (85.37)	43 (93.48)		
Obscured	6 (14.63)	3 (6.52)		

Superior mediastinum			1.84 (1.23–2.73)	0.013
Normal	29 (70.73)	42 (91.30)		
Abnormal	12 (29.27)	4 (8.70)		

Heart shadow				
Normal	34 (82.93)	42 (91.30)		
Widened	7 (17.07)	4 (8.70)	1.42 (0.85–2.37)	0.241

**Table 3:** Chest X-ray and categorical MEWS as predictor of mortality.

Mortality predictor	Mortality		Sensitivity	Specificity	PPV (%)	NPV (%)
	Dead *N* = 41	Alive *N* = 46				
Chest X-ray status						
Abnormal	34	30				
Normal	7	16	82.93	34.78	53.13	69.57

MEWS						
<5	2	11				
≥5	37	36	90.24	23.40	50.68	84.62

## Abbreviations

MEWS: Modified Early Warning Score
ICU: Intensive Care Unit
CXR: Chest X-ray
GCS: GLASCOW coma scale
MNRTH: Mulago National Referral and Teaching Hospital.
